# Artificial Intelligence in Fractured Dental Implant Detection and Classification: Evaluation Using Dataset from Two Dental Hospitals

**DOI:** 10.3390/diagnostics11020233

**Published:** 2021-02-03

**Authors:** Dong-Woon Lee, Sung-Yong Kim, Seong-Nyum Jeong, Jae-Hong Lee

**Affiliations:** 1 Department of Periodontology, Veterans Health Service Medical Center, Seoul 05368, Korea; dongden@daum.net; 2 Department of Prosthodontics, Veterans Health Service Medical Center, Seoul 05368, Korea; ksungy@hotmail.com; 3 Department of Periodontology, Daejeon Dental Hospital, Institute of Wonkwang Dental Research, Wonkwang University College of Dentistry, Daejeon 35233, Korea; seongnyum@wku.ac.kr

**Keywords:** artificial intelligence, dental implants, deep learning, supervised machine learning

## Abstract

Fracture of a dental implant (DI) is a rare mechanical complication that is a critical cause of DI failure and explantation. The purpose of this study was to evaluate the reliability and validity of a three different deep convolutional neural network (DCNN) architectures (VGGNet-19, GoogLeNet Inception-v3, and automated DCNN) for the detection and classification of fractured DI using panoramic and periapical radiographic images. A total of 21,398 DIs were reviewed at two dental hospitals, and 251 intact and 194 fractured DI radiographic images were identified and included as the dataset in this study. All three DCNN architectures achieved a fractured DI detection and classification accuracy of over 0.80 AUC. In particular, automated DCNN architecture using periapical images showed the highest and most reliable detection (AUC = 0.984, 95% CI = 0.900–1.000) and classification (AUC = 0.869, 95% CI = 0.778–0.929) accuracy performance compared to fine-tuned and pre-trained VGGNet-19 and GoogLeNet Inception-v3 architectures. The three DCNN architectures showed acceptable accuracy in the detection and classification of fractured DIs, with the best accuracy performance achieved by the automated DCNN architecture using only periapical images.

## 1. Introduction

Dental implants (DIs) have shown a high survival and success rate, making them an indispensable and predictable treatment modality for restoring missing teeth [1]. In a recent systematic review of DI rehabilitation outcomes, the 10-year survival rate was reported as 96.4% (95% CI = 95.2–97.5%), and the overall cumulative survival rate for a follow-up study of 15 years was reported as 82.6%, respectively [1,2]. Accordingly, various biological (including peri-implant mucositis and peri-implantitis) and mechanical (including chipping, screw loosening and fractures, and ceramic and fixture fractures) complications could increase and require a multiplicity of re-interventions [3].

Among mechanical complications, fracture of DI is almost impossible to repair or modify; therefore, it is one of the critical causes for the possibility of DI failure and explantation. Biomechanical and physiological overload and stress with non-passive prosthesis fit might be considered to be the most common risk factors for DI fracture [4,5]. As shown in recent studies, various clinical variables (including age, sex, diameter, length, placement position, with or without bone graft, fixture material (CP4 or alloy), polished or unpolished cervical feature, butt or conical abutment connection, micro- or macro- thread, and platform switching) may affect the fracture of DIs, and the diameter, position, history of bone graft, and micro-thread presence of the DI are significantly related to the occurrence of DI fractures [6,7]. In a systematic review of long-term results of more than 5 years, the ratio of fracture was reported as 0.18%, and a recent 12-year follow-up study showed a frequency of 0.92% in 19,006 fractured DIs of 5125 patients [6,7]. Since the prevalence and incidence of fracture is relatively rare and often asymptomatic, it is a very difficult and challenging task for early detection in actual clinical practice. When DI fracture is undiagnosed or diagnosed late, post-traumatic and inflammatory reactions that induce severe bone loss around DI will inevitably occur [7].

Artificial intelligence, specifically deep learning and neural network-related technologies, has developed significantly over the last 10 years and is now widely applied in the medical and dental fields [8,9]. Deep convolutional neural networks (DCNNs) are a branch of deep learning methods that use a cascade of multiple layers of nonlinear transformation to generate high-level abstraction, thereby increasing its versatility for identifying representative patterns or features [10,11]. Recently, DCNN has expanded in popularity and has become the cutting-edge technology for medical image analysis, including detection, segmentation, and classification [12].

In orthopedic and trauma surgery, DCNN has been successfully used to detect and classify various types of human bone fractures, and in particular, has shown excellent accuracy performance of diagnosing hip, proximal humerus, ankle, and femur fractures [13,14,15,16]. In dentistry practice, one study was recently conducted to improve the detection accuracy of vertical root fractures based on dental radiographic images, but as far as we are aware, there is no research related to DI fracture [17]. Therefore, the aim of this study is to evaluate the reliability and validity of deep learning for detection and classification of DI facture based on three different DCNN architectures using panoramic and periapical radiographic images.

## 2. Materials and Methods

The study design and protocol were reviewed and authorized by the Institutional Review Board of the Veterans Health Service Medical Center (VHSMC, approval no. BOHUN 2020-03-012-001, 13 April 2020) and Daejeon Dental Hospital, Wonkwang University (approval No. W2011/002-001, 23 April 2020), and the need for informed or written consent was waived as part of the study approval. This study was conducted in compliance with the revised Declaration of Helsinki and followed the STROBE guidelines for the conduct and reporting of observational studies [18,19].

### 2.1. Dataset

We retrospectively obtained a dataset from January 2006 to December 2015 in VHSMC and from April 2007 to December 2019 in WKUDH. A total of 21,398 DIs in 7281 patients were reviewed through dental electronic records, clinical photos, and dental digital radiographic images by two participating board-certified periodontists (DWL and JHL) and one board-certified prosthodontist (SYK). All periapical images were obtained using the standard paralleling technique, and radiographic images with severe noise, haziness, or distortion were excluded by the three dental professionals mentioned. Following this, one periodontist (JHL) manually and multiply segmented the anonymized DICOM format DI images (panoramic images with a pixel resolution of 2868 × 1504 and periapical images with a pixel resolution of 1876 × 1402), using radiographic image analysis software (INFINITT PACS, INFINITT Healthcare and Osirix X 10.0 64-bit version, Pixmeo SARL), into the region of interest. Finally, 251 intact and 198 fractured DIs were identified and included as the total dataset in this study. The fractured DIs were classified into three groups, referring to a previous study that analyzed the pattern of fracture (Type I, horizontal and vertical fractures limited within and around the crestal module; Type II, vertical fracture beyond the crestal module; and Type III, horizontal fracture over the crestal module) [20]. However, the number of type-III fractured DIs was very small (n = 4) in the process of obtaining datasets; therefore, only type-I and -II fractured DIs were included in this study. The details and numbers of the panoramic and periapical images for each intact and fractured DI are shown in Table 1.

### 2.2. Preprocessing

All included radiographic images were resized to 224 × 224 pixels for the VGGNet-19 architecture, 299 × 299 pixels for the GoogLeNet Inception v3 architecture, and 224 × 224 pixels for the automated DCNN architecture, respectively. The dataset was randomly divided into 60% training, 20% validation, and 20% test datasets for model development and accuracy performance predictions. The preprocessing includes pixel normalization, and one-hot encoding was deployed to reduce irregularities in the dataset. The training dataset was randomly augmented 100 times using rotation (range of 30°), width and height shifting (range of 0.2), zooming (range of 0.2), and horizontal and vertical flip. No augmentation procedure was performed in the validation and test datasets.

### 2.3. Architecture of the DCNN

**Figure 1:**
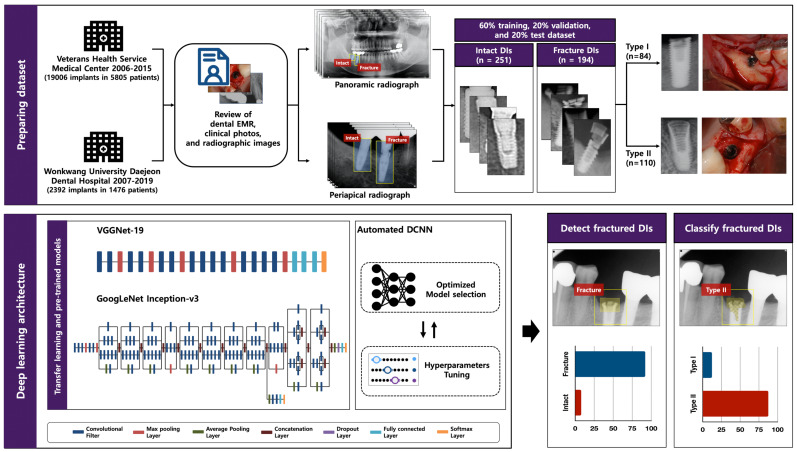
Schematic illustration of deep convolutional neural network (DCNN) applications. Dataset prepared from anonymized raw panoramic and periapical radiographic images, and all included dental implants (DIs) were manually cropped and labeled. Training process was based on three different DCNN architectures to compare accuracy performance to detect and classify types of fractured DIs.

We conducted a training process based on three different DCNN architectures, to compare the accuracy performance to detect and classify the types of fractured DIs (Figure 1):

- The VGGNet-19 architecture is a 19-layer DCNN model for the 2014 ImageNet Large Scale Visual Recognition Challenge (ILSVRC) competition with a 7.3% Top-5 error rate, by the Visual Geometry Group at the University of Oxford [21].
- The GoogLeNet Inception-v3 architecture, which showed excellent performance in the 2014 ILSVRC competition with a 6.7% Top-5 error rate, consists of 22 deep layers and 9 inception modules [22].
- The automated DCNN architecture was designed to search for optimized DCNN model selection and efficient hyperparameter tuning (including number of convolutional layers, learning rate, dropout rate, batch size, number of epochs, and optimizer type) [23]. All automated DCNN analyses were conducted using the Neuro-T version 2.1.1 (Neurocle Inc., Seoul, Korea).

The VGGNet-19 and Inception-v3 architectures utilized the transfer learning and pre-trained model with weights from approximately 1.28 million images (ImageNet) and 11,980 DI images of datasets we have built in the past [24]. For training the VGGNet-19 and Inception-v3 models, the top layers were truncated by defining a new fully connected softmax classification and output layer with a practical number of categories. We implemented the stochastic gradient descent (SGD) algorithm and used the Adam optimizer with an initial learning rate of 0.0001 and a decay rate of 0.001 based on the Keras application programming interface in the Python [25]. The models were trained for a maximum of 2000 epochs with a dropout probability of 0.5 during training to avoid overfitting. The final models were chosen as the pre-trained architectures with the best performance on the validation datasets. The automated DCNN architecture automatically created effective deep learning models and searched for the optimal hyperparameters during training and inference. The final automated model consisted of 18 layers with no dropout, with an Adam optimizer and L2 normalization. The batch size was set to 10, and epochs were set to 25.

## 3. Results

### 3.1. Detection of Fractured DIs

**Figure 2:**
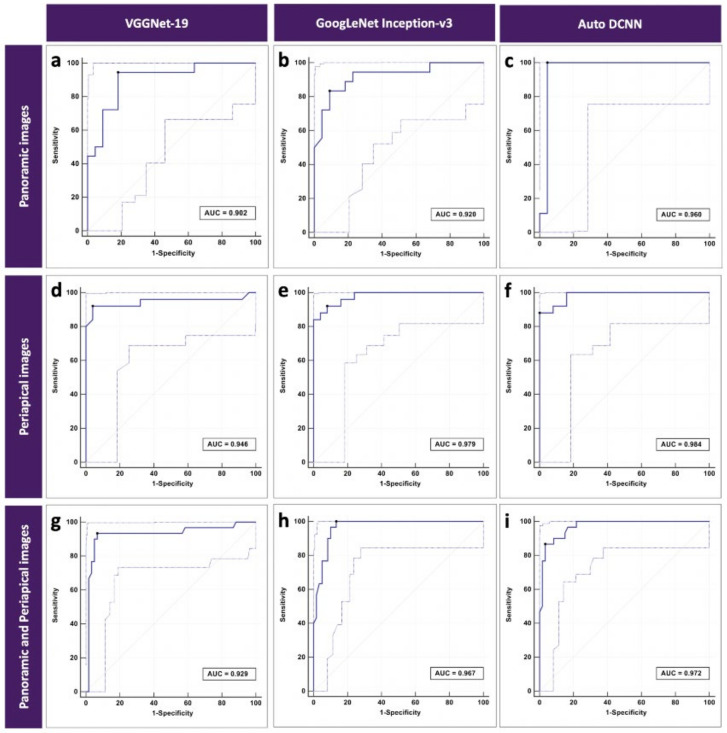
Receiver operating characteristic (ROC) curves for detection of fractured Dis consisting of (**a–c**) 40 panoramic images, (**d**–**f**) 49 periapical images, and (**g**–**i**) 89 panoramic and periapical images. Plots include 95% confidence bounds.

The automated DCNN architecture achieved the best accuracy performance using periapical images, with the highest AUC of 0.984 (95% CI = 0.900–1.000), sensitivity of 0.880, specificity of 1.000, and Youden index of 0.880. The fine-tuned and pre-trained VGGNet-19 architecture achieved the lowest accuracy performance using panoramic images, with an AUC of 0.902 (95% CI = 0.765–0.973), sensitivity of 0.944, specificity of 0.818, and Youden index of 0.762. The detection accuracy of the fractured DIs is shown in detail in Table 2. Figure 2 shows the ROC curves of three different DCNN architectures using only panoramic, only periapical, and panoramic and periapical images.

### 3.2. Classification of Types of Fractured DIs

**Figure 3:**
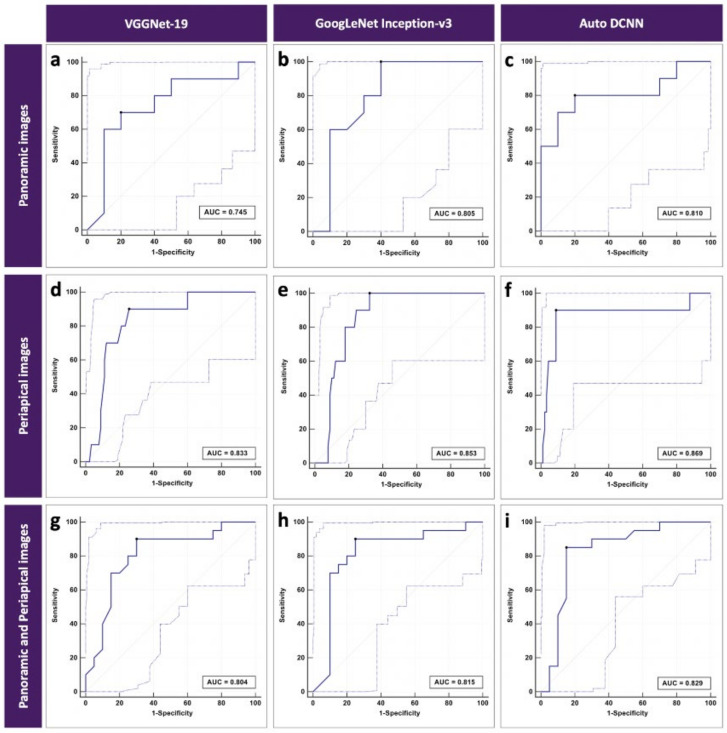
ROC curves for classification of types of fractured Dis consisting of (**a**–**c**) 19 panoramic images, (**d**–**f**) 20 periapical images, and (**g**–**i**) 39 panoramic and periapical images. Plots include 95% confidence bounds.

The automated DCNN architecture achieved the highest accuracy performance using periapical images, with the highest AUC of 0.869 (95% CI = 0.778–0.929), sensitivity of 0.900, specificity of 0.911, and Youden index of 0.811. The fine-tuned and pre-trained Inception-v3 achieved the second-highest accuracy performance using periapical images, with an AUC of 0.853 (95% CI = 0.769–0.916), sensitivity of 1.000, specificity of 0.677, and Youden index of 0.677. The VGGNet-19 architecture achieved the lowest accuracy performance using panoramic images, with an AUC of 0.745 (95% CI = 0.504–0.910), sensitivity of 0.700, specificity of 0.800, and Youden index of 0.500. The classification accuracy of the fractured DIs is shown in detail in Table 3. Figure 3 shows the ROC curves of all three different DCNN architectures using only panoramic, only periapical, and panoramic and periapical images.

## 4. Discussion

Artificial intelligence and deep learning are progressing and expanding rapidly, and have shown promising applications for dental image analysis in recent years. In particular, as newly developed DCNN models and algorithms are continuously adopted and coupled with the area of implant dentistry, it may be an important adjunct for diagnosis, treatment, and prognosis assessments [26]. Recent DCNN-related studies confirmed that various types of DIs with different shapes, lengths, or dimensions can be effectively detected and classified using panoramic and periapical images [27,28,29].

Automated DCNN architecture that automatically finds well-performing and specialized models and optimal hyperparameters is receiving increasing attention in the field of computer science, but research based on automated DCNN architecture in the medical and dental fields is quite insufficient [30,31]. Our most recent research showed that the automated DCNN architecture was highly accurate (AUC = 0.954, 95% CI = 0.933–0.970) for classifying similar shapes of six different morphological types of DIs based on panoramic and periapical images, and achieves better classification accuracy performance (AUC = 0.961, 95% CI = 0.941–0.976) compared to most of the 25 participating dental professionals, including board-certified periodontists, periodontal residents, and residents not specialized in periodontology [24].

The VGGNet-19 and GoogLeNet Inception-v3 architectures, with transfer learning and fine-tuning of pretrained weights, are already being actively used and show highly consistent and predictable outcomes in the fields of periodontology, restorative dentistry, and oral surgery [32,33,34]. All three deep learning algorithms applied in the current study achieved a fractured DI detection accuracy of over 0.90 AUC, and in particular, automated DCNN using periapical images showed the best accuracy performance (AUC = 0.984, 95% CI = 0.900–1.000), compared to the modified VGGNet-19 (AUC = 0.946, 95% CI = 0.842–0.990) and GoogLeNet Inception-v3 (AUC = 0.979, 95% CI = 0.892–0.999) architectures.

It is difficult to accurately classify similar shapes, but different types of fractured DI can be examined through dental radiography, and considerable clinical experience is required for proper type classification of DI fractures. Except for the VGGNet-19 architecture (AUC = 0.745, 95% CI = 0.504–0.910) using panoramic images, included DCNN architectures achieved a classification accuracy of over 0.80 AUC, and in particular, automated DCNN architecture using periapical images showed the highest and most reliable classification accuracy performance (AUC = 0.869, 95% CI = 0.778–0.929). However, although a total of 21,398 DIs were reviewed in 7281 patients from two dental hospitals, only four radiographic images were classified as type III. Therefore, type-III DI fractures were excluded from the dataset and are considered one of the drawbacks of the current study.

Regardless of the type of dataset (including panoramic-only, periapical-only, and panoramic and periapical images) used for DCNN model training, our previous studies confirmed that there is no statistically significant difference in accuracy performance for the identification of DIs [24,29]. The results of this study, consistent with previous studies, indicated that the classification accuracy was not significantly different among the use of panoramic-only, periapical-only, and panoramic and periapical image datasets based on three different DCNN architectures. However, regardless of the type of DCNN architecture, when periapical-only images were used as a dataset, it consistently showed the highest accuracy on average. This is owing to the fact that the periapical image has a higher resolution and sharpness than the panoramic image, and therefore it is expected that the use of the periapical images as a dataset will be more effective in improving the detection and classification of fractured DIs.

The current study has several limitations and future directions that need to be considered. First, because the prevalence and incidence of DI fracture are very low, it is not easy to obtain more than a significant number of fractured DI image datasets. In this study, although more than 20,000 radiographic images were reviewed at two dental hospitals, only 194 fractured DI radiographic images were included in the dataset. Collecting a larger quantity and quality dataset through more dental hospitals is considered the most important prerequisite for clinical use in the field of implant dentistry. Second, the use of low-resolution image datasets for training and validating the DCNN architecture is another limitation of this study. Owing to the limitation of available resources, including computing power storage capacity, we used reduced low-resolution panoramic and periapical images cropped and resized. Additional studies are necessary to confirm whether higher accuracy performance could be achieved by using a high-resolution image dataset.

## 5. Conclusions

In accordance with the limited results obtained from this study, VGGNet-19, GoogLeNet Inception-v3, and automated DCNN architectures showed acceptable accuracy outcomes in the detection and classification of fractured DIs, with the best performance achieved by the automated DCNN architecture using only periapical radiographic images. Further prospective and clinical evidence is necessary to determine the feasibility of applying DCNN architecture in dental practice.

## Figures and Tables

**Table 1:** Number of panoramic and periapical radiographic images for intact and fractured dental implants (DIs). Dataset collected from two dental hospitals: Veterans Health Service Medical Center and Daejeon Dental Hospital, Wonkwang University.

	Dataset	
	Frequency	Percentage
Intact DIs		
Panoramic images	110	43.8
Periapical images	141	56.2
Fractured DIs, Type I		
Panoramic images	41	48.8
Periapical images	43	51.2
Fractured DIs, Type II		
Panoramic images	52	47.3
Periapical images	58	52.7

Fractured DIs were classified as follows: Type I, horizontal and vertical fractures limited within and around crestal module of implant fixture; Type II: vertical fracture beyond crestal module of implant fixture.

**Table 2:** Detection accuracy of fractured DIs between three different DCNN architectures.

Variables	AUC	95% CI	SE	Sensitivity	Specificity	Youden Index
Panoramic images						
VGGNet-19	0.902	0.765–0.973	0.049	0.944	0.818	0.762
GoogLeNet Inception-v3	0.920	0.790–0.982	0.045	0.833	0.909	0.742
Automated DCNN	0.960	0.845–0.997	0.040	1.000	0.954	0.954
Periapical images						
VGGNet-19	0.946	0.842–0.990	0.039	0.920	0.960	0.880
GoogLeNet Inception-v3	0.979	0.892–0.999	0.014	0.920	0.920	0.840
Automated DCNN	0.984	0.900–1.000	0.012	0.880	1.000	0.880
Panoramic and periapical images						
VGGNet-19	0.929	0.854–0.972	0.037	0.933	0.933	0.866
GoogLeNet Inception-v3	0.967	0.906–0.993	0.015	1.000	0.866	0.866
Automated DCNN	0.972	0.913–0.995	0.014	0.866	0.966	0.833

DCNN, deep convolutional neural network; AUC, area under the curve; CI, confidence interval; SE, standard error.

**Table 3:** Classification accuracy of types of fractured DIs between different three DCNN architectures.

Variables	AUC	95% CI	SE	Sensitivity	Specificity	Youden Index
Panoramic images						
VGGNet-19	0.745	0.504–0.910	0.122	0.700	0.800	0.500
GoogLeNet Inception-v3	0.805	0.569–0.945	0.110	1.000	0.600	0.600
Automated DCNN	0.810	0.575–0.948	0.106	0.800	0.800	0.600
Periapical images						
VGGNet-19	0.833	0.745–0.900	0.058	0.900	0.744	0.644
GoogLeNet Inception-v3	0.853	0.769–0.916	0.040	1.000	0.677	0.677
Automated DCNN	0.869	0.778–0.929	0.085	0.900	0.911	0.811
Panoramic and periapical images						
VGGNet-19	0.804	0.648–0.912	0.074	0.900	0.700	0.600
GoogLeNet Inception-v3	0.815	0.661–0.920	0.077	0.901	0.749	0.650
Automated DCNN	0.829	0.677–0.929	0.072	0.850	0.850	0.700

DCNN, deep convolutional neural network; AUC, area under the curve; CI, confidence interval; SE, standard error.

## Data Availability

Data sharing not applicable.
